# Measles—Clinical and Biological Manifestations in Adult Patients, Including a Focus on the Hepatic Involvement: Results from a Single-Center Observational Cohort Study from Romania

**DOI:** 10.3390/jcm13185535

**Published:** 2024-09-18

**Authors:** Victoria Bîrluțiu, Rares-Mircea Bîrluțiu

**Affiliations:** 1 Faculty of Medicine, Lucian Blaga University of Sibiu, Str. Lucian Blaga, Nr. 2A, 550169 Sibiu, Romania; 2 Infectious Diseases Department, County Clinical Emergency Hospital, Bvd Corneliu Coposu, Nr. 2–4, 550245 Sibiu, Romania; 3 Faculty of Medicine, University of Medicine and Pharmacy “Carol Davila”, 050474 Bucharest, Romania; 4 Clinical Hospital of Orthopedics, Traumatology, and Osteoarticular TB Bucharest, B-dul Ferdinand 35–37, Sector 2, 021382 Bucharest, Romania

**Keywords:** measles, hepatic involvement, clinical manifestations, adult patients, respiratory failure, clinical forms

## Abstract

**Background**: Hepatic involvement in measles, particularly among adolescents and adults, has been recognized since 1960. This involvement typically manifests during the eruptive phase of the disease and is primarily characterized by hepatocellular dysfunction, with jaundice being a less common occurrence. Studies have reported hepatic involvement in 80–86% of measles cases among young adults associated with severe forms of the disease with intra-infectious hepatitis. Recent data from Romania indicated 20,035 confirmed measles cases between January and June 2024, including 17 fatalities and significant hepatic alterations. These findings underscore the need for the further investigation of the hepatic manifestations of measles. The primary objective of our study was to evaluate the clinical and baseline characteristics of the enrolled patients with a brief assessment of liver impairment. **Methods**: In light of these observations, we conducted a retrospective analysis between 1 November 2023 and 15 June 2024 in patients aged >16 years who were confirmed, by the detection of measles IgM in serum samples, to have acute measles infection and hospitalization. **Results**: During the study period, 71 hospitalized patients were diagnosed with measles, of whom 37 were female (52.1%), with ages ranging from 16 to 64 years (mean age 34.21 years). Most cases (77.5%) exhibited moderate clinical forms of measles, while 22.5% had severe forms. Respiratory failure requiring oxygen therapy was uncommon (25.4% of severe pneumonia cases). Although a Pearson chi-square test indicated no significant association between the presence of pneumonia and clinical form (*p* = *0.066*), the likelihood ratio test suggested a potential link (*p* = 0.018). Hepatic involvement was common with elevated AST (87.3%) and ALT (76%) levels. Jaundice was observed in 12.7% of patients. GGT changes were noted in 35.2% of cases, with significant correlations between GGT levels and disease severity (*p* = *0.001*). Analysis of various symptoms revealed significant associations between nausea, dyspnea, and severe clinical forms. Anorexia, diarrhea, and nausea were the most frequently reported symptoms. Thrombocytopenia was observed in 11 patients, with no significant correlation with disease severity. Comorbidities, such as COPD, were significantly associated with disease severity (*p* = *0.010*). **Conclusions**: Our findings highlight that, while cytolytic hepatic damage is a typical response to measles infection, cholestatic involvement may serve as an indicator of more severe disease progression.

## 1. Introduction

Hepatic involvement in measles has been documented since 1960 in the Berry TJ study, particularly in adolescents and adults [1]. This involvement is associated with the eruptive phase of the disease, resulting primarily from hepatocellular dysfunction and much less frequently from jaundice.

Gavish et al. (1983) described hepatic involvement in 80% of 65 measles cases in young adults, with only 5 cases presenting with jaundice. The authors associated severe forms of measles with the presence of intra-infectious hepatitis and monitored resolution to complete recovery over a 7-year follow-up period, correlating progression with the occurrence of bacterial superinfections [2]. In another study, Giladi et al. analyzed 291 cases of adult measles and found hepatic involvement in 86% of these cases, considering it a characteristic of the disease rather than a complication [3]. Leibovici conducted a clinical and paraclinical analysis of 461 young patients with measles and found elevated liver enzyme levels in 51% of the cases [4].

Dinh et al. conducted a study in France from 2010–2011 on 80 patients aged between 23.5 and 34 years, highlighting hepatocellular syndrome in 65% of cases and hyperbilirubinemia in only 4% of cases [5]. Biron et al., during the Nantes epidemic in 2008–2009, described hepatocellular damage or cholestasis in 9 out of 11 adults, with rapid normalization [6].

Sternfeld et al. (2010) described a case of liver transplant rejection triggered by an acute measles infection. This case involved a 31-year-old male who received a liver graft at the age of 18 years for severe Wilson’s disease and was on immunosuppressive therapy with tacrolimus. Rejection, confirmed by histopathological changes, including portal inflammation and endotheliitis, was successfully treated with methylprednisolone at 1000 mg four times daily for three days [7]. Satoh et al. investigated hepatic changes during measles (elevated transaminases and LDH) infection through liver biopsy, revealing necrotic changes without portal involvement, and detected viral RNA via RT-PCR, suggesting direct action on hepatocytes [8].

Nobili described a case of fulminant hepatitis in an 18-month-old during measles infection, with AST levels peaking at 50,800 U/L, ALT at 1261 U/L, and a prothrombin time of 15.8%. Histopathological examination revealed diffuse perivenular necrosis involving 60% of hepatocytes, and electron microscopy confirmed the presence of the measles virus both intracellularly and intranuclearly. The patient was successfully treated with left-lobe transplantation from a young donor [9]. Another case of acute liver failure was described in a 6-month-old female infant with lethargy, hepatomegaly, and mild jaundice (total bilirubin 1.7 mg/dL) during measles infection, with AST at 7980 U/L, ALT at 5120 U/L, and a prothrombin time of 15%. The patient showed favorable outcomes with supportive treatment and fresh plasma, vitamin K, and N-acetylcysteine [10].

Hepatic involvement appears to be more common in Europe and America, whereas in Japan, increases in LDH levels are more frequently observed than elevations in transaminase levels [11].

Between 1 January 2024 and 30 June 2024, Romania reported 20,035 confirmed measles cases, including 17 associated deaths. In Sibiu County alone, 723 cases were documented, corresponding to a morbidity rate of 185.29 per 100,000 inhabitants, according to data from the National Institute of Public Health [12].

The emergence of a higher number of cases that were also associated with significant hepatic alterations was the observation that prompted an in-depth investigation into hepatic involvement in measles.

The primary objective of our study was to evaluate the clinical and baseline characteristics of the enrolled patients, with a brief, focused assessment of liver impairment in these cases. The secondary objective was to identify the risk factors associated with distinct clinical forms of the disease.

## 2. Materials and Methods

After confirming a significant number of measles cases in the adult population, a single-center observational cohort study on patients diagnosed with measles and hospitalized at the Sibiu Clinical County Hospital, Romania was performed. In these analyses, we retrospectively included the data of all consecutive patients aged >16-years-old, who were confirmed by the detection of measles IgM in serum samples to have acute measles infection, admitted to the Infectious Diseases Clinic of Sibiu Clinical Country Hospital, Romania between 1 November 2023 and 15 June 2024.

The medical records of the enrolled patients included demographic characteristics, risk factors and comorbidities, laboratory investigations, treatments, clinical forms of the disease, and complications. Both authors assessed data retrieval and consistency.

We considered any values above normal for aspartate aminotransferase (AST), alanine aminotransferase (ALT), lactate dehydrogenase (LDH), and total bilirubin (BT) as indicative of hepatic involvement, provided that there were no other attributable causes, such as hepatotropic viruses, chronic hepatic diseases, or toxic, metabolic, autoimmune, or congenital conditions. Major hepatic involvement was defined as the AST or ALT levels reaching five times the upper limit of normal (5× ULN). We categorized cases with hepatic involvement into the following groups based on enzyme elevation levels: 1–2× ULN, 3–5× ULN, 6–10× ULN, 11–15× ULN, and >16× ULN. The same categorization was applied to evaluate cholestasis syndrome using gamma-glutamyl transferase (GGT) levels.

In our study, the clinical forms of measles were defined based on the severity of pneumonia and the presence of respiratory failure, necessitating various forms of oxygen therapy based on the classification used for SARS-CoV-2 infection. Moderate forms of the disease included patients with imaging-confirmed pneumonia but without hypoxemia (in the absence of pre-existing respiratory conditions prior to the current illness), while the severe form was represented by patients with respiratory distress, with SaO_2_ < 94% on room air and imaging abnormalities indicating pulmonary involvement.

Statistical analyses were performed using the IBM SPSS Statistics version 29. Continuous variables were summarized using univariate descriptive statistics, reporting counts and percentages where applicable. The Mann–Whitney test was used to compare skewed distributions of continuous variables, whereas categorical variables were assessed using the chi-square, Fisher’s exact, or Cramer’s V tests as appropriate. The normality of data distribution was evaluated using both Kolmogorov–Smirnov and Shapiro–Wilk tests. Statistical significance was set at *p* < *0.05*.

Written informed consent was obtained from all subjects involved in the study.

Our study was conducted in accordance with the principles of the Declaration of Helsinki and was approved by the Institutional Ethics Committee (approval number 16844/04.07.2024); they also encouraged publishing the article.

## 3. Results

Of the hospitalized patients in our clinic between 1 November 2023 and 15 June 2024, 71 patients were diagnosed with measles and required hospitalization, and all included cases in this analysis had a complete electronic medical record. Of the enrolled patients, 37 (52.1%) were female. Their ages ranged from 16 to 64 years, with a mean age of 34.21 years and std. deviation of 11.57 years. Female patients enrolled in our study had a higher mean age (36.05 years), with a standard deviation of 11.15 years, compared to male patients (32.21 years), with a standard deviation of 11.85 years, suggesting that the female group was slightly older on average.

In terms of the vaccination status of the enrolled patients in our study with either measles or with the combined measles/mumps/rubella (MMR) vaccine, 28 patients reported no vaccination. Forty-three patients had a complete vaccination status for measles.

In terms of the clinical presentation at the time of hospital admission, 66 out of 71 patients exhibited pyrexia, 60 presented with a maculopapular exanthem, 64 demonstrated oculonasal catarrh, 12 displayed Koplik’s spots, and 64 had a persistent cough.

In our study, we identified several notable clinical features in the progression of measles among adults. Specifically, in 24 cases, we observed the onset of the rash localized to the proximal upper extremities on the second day of the exanthem, which coincided with centrifugal spread to the thorax and further intensification by the third day. In 43 cases, the rash became confluent across the face, thorax, and abdomen, resulting in extensive areas of confluence with minimal spared skin. In eight cases, the exanthem progressed to a hemorrhagic-purpuric form. Additionally, we documented the persistence of Koplik’s spots during the exanthematous phase for a duration of 48–72 h in eight cases.

In terms of the clinical forms of the hospitalized patients, the majority of cases, 55 (77.5%), had a moderate clinical form of measles and a smaller proportion, 16 (22.5%), of cases had a severe clinical form. Most of the enrolled patients, 53 (74.6%), did not have respiratory failure requiring different forms of oxygen therapy.

Analysis of the data revealed that, in the overall study population, there was a significant association between the presence of pneumonia (43 cases) and respiratory failure requiring different forms of oxygen therapy (chi-square value = 3.953, *p* = *0.047*). Of the enrolled patients who also presented with respiratory failure requiring different forms of oxygen therapy, 25.4% of the cases had severe pneumonia and respiratory failure requiring different forms of oxygen therapy, whereas among moderate clinical form cases, only a small proportion (2 out of 45) with pneumonia who were already on oxygen therapy prior to the admission for their underlying conditions also experienced respiratory failure requiring different forms of oxygen therapy. Correlation measures for the overall sample, including Pearson’s R and Spearman’s correlations, also indicated a weak but significant positive correlation (0.236, *p* = *0.048*), supporting the association between pneumonia and respiratory failure requiring different forms of oxygen therapy.

The presence of pneumonia in patients with moderate and severe clinical forms of the disease was present in our study in 45 and 16 cases, respectively. The statistical analysis of our data using cross-tabulation revealed that there was no statistically significant association between the presence of pneumonia and the clinical form (moderate or severe) at the conventional 5% significance level. This conclusion was supported by the Pearson chi-square test, which yielded a *p*-value of *0.066*, which was slightly above the threshold of *0.05*. Additional tests, including the continuity correction and Fisher’s exact test, with *p*-values of *0.152* and *0.103* (two-sided), respectively, further corroborated the lack of a significant association. However, the likelihood ratio test indicated a significant association, with a *p*-value of *0.018*, suggesting a potential link between the presence of pneumonia and its clinical form. Furthermore, both Pearson’s R and Spearman’s rank correlation coefficients, which were *0.218* with a significance level of *0.067*, suggested a weak positive correlation that was marginally non-significant.

An overview of the clinical characteristics and laboratory tests performed during the hospitalization period of the enrolled patients is shown in the following table (Table 1).

In terms of frequency distribution, bilirubin levels were above the reference values. Most patients, 62 (87.3%), had bilirubin levels above the reference values. A small proportion of the patients, 9 (12.7%), had bilirubin levels above the reference values.

Cross-tabulations between laboratory-performed tests, reported in Table 1, were also performed. Statistical analysis highlighted no significant associations between AST and ALT levels, WBCs count, neutrophil count, lymphocyte count, total bilirubin level, or clinical form (moderate or severe). Additionally, there were no significant correlations between AST and ALT levels, WBCs count, neutrophil count, lymphocyte count, total bilirubin level, and clinical form.

Thrombocytopenia in our group of patients was encountered in 11 cases, ranged between 89,000–149,000 platelets (PLTs)/μL, with a mean of 124,272.72 PLTs/μL and a standard deviation of 20,895.41 PLTs/μL. Statistical analysis indicated no significant association between the occurrence of low platelet counts and the clinical form (moderate or severe) of the disease, based on the Pearson chi-square test (*p* = *0.682*), continuity correction (*p* = *0.987*), likelihood ratio test (*p* = *0.688*), and Fisher’s exact test (*p* = *0.702*). Pearson’s R and Spearman’s correlations also showed weak, non-significant correlations.

The results of our statistical analysis suggested a significant linear association between the neutrophil-to-lymphocyte (NL) ratio and clinical form (moderate or severe), based on the linear-by-linear association test (*p* = *0.048*). This was further supported by Pearson’s R and Spearman’s correlations, both of which showed statistically significant correlations (*p* = *0.047* and *p* = *0.029*, respectively). Although the Pearson chi-square and likelihood ratio tests indicated no significant association at the 5% level (*p* = *0.170* and *p* = *0.089*, respectively), the close values suggested a potential trend.

In addition, based on the cross-tabulations performed, there was a significant linear association between gamma-glutamyl transferase (GGT) levels and clinical form (moderate or severe), based on the linear-by-linear association test (*p* = *0.001*) and the likelihood ratio test (*p* = *0.049*). Pearson’s R test indicated a statistically significant moderate positive correlation (*p* = *0.001*). Although Pearson’s chi-square test (*p* = *0.109*) and Spearman’s correlation (*p* = *0.080*) did not indicate a statistically significant association at the 5% level, they suggested a potential trend.

**Figure 1:**
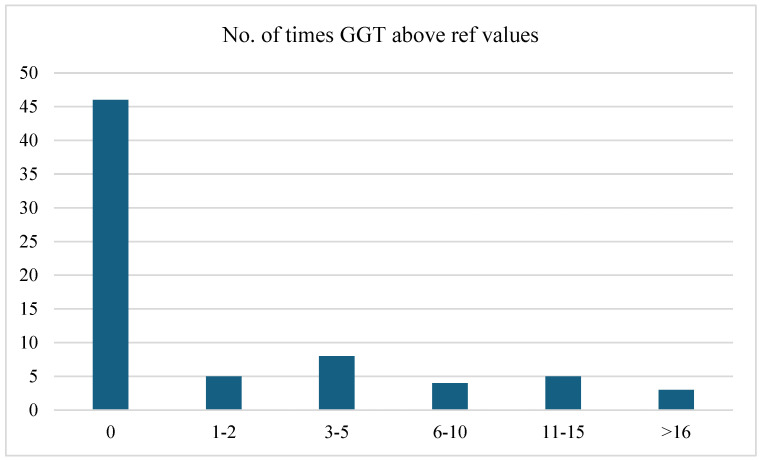
Frequency distribution of the number of times GGT levels were above reference values.

Figure 1 highlights the frequency distribution of the number of times gamma-glutamyl transferase (GGT) levels were above the reference values. This distribution indicated that most patients (64.8%) maintained GGT levels within the reference range. A small proportion of patients experienced elevated GGT levels, with the highest frequency (11.3%) being 3–5 times above the reference range. Statistical analysis indicated that there was no significant association between the frequency of GGT levels being above the reference values and the clinical form (moderate or severe), based on the Pearson chi-square test (*p* = *0.055*). The likelihood ratio test (*p* = *0.090*) supported this conclusion, although both tests suggested a trend towards significance.

**Figure 2:**
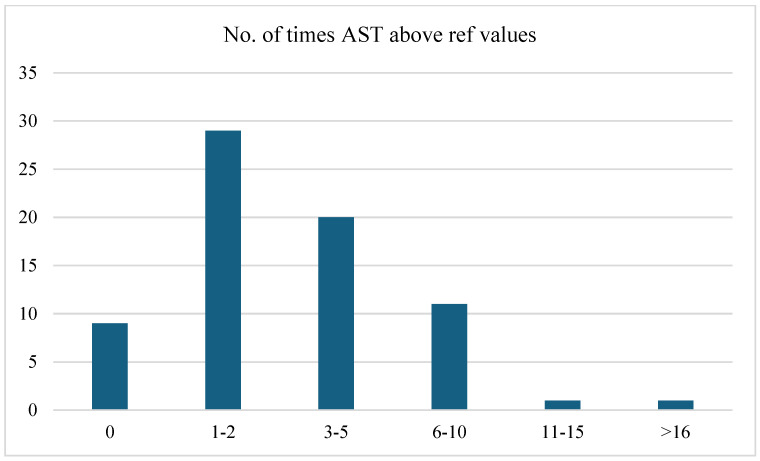
Frequency distribution of the number of times AST levels were above reference values.

Figure 2 highlights the frequency distribution of aspartate aminotransferase (AST) levels above the reference values. This distribution indicated that a significant proportion of patients (40.8%) experienced AST elevations 1–2 times higher than the reference values. A notable percentage of patients (28.2%) had AST levels elevated 3–5 times, whereas 15.5% had elevations of 6–10 times. Fewer patients maintained AST levels within the reference range (12.7%) or had elevations >10 times. Statistical analysis indicated that there was no significant association between the frequency of AST levels above the reference values and the clinical form (moderate or severe), based on the Pearson chi-square test (*p = 0.129*). The likelihood ratio test (*p* = *0.063*) suggested a trend towards significance, but this was not conclusive.

**Figure 3:**
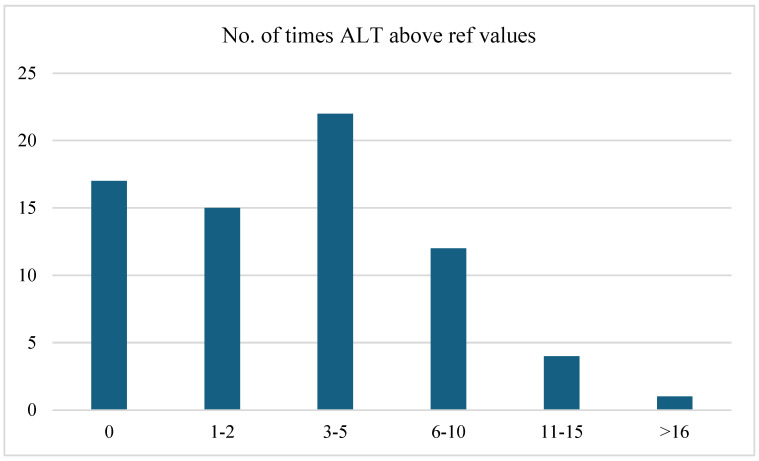
Frequency distribution of the number of times ALT levels were above reference values.

Figure 3 highlights the frequency distribution of the number of times alanine aminotransferase (ALT) levels were above the reference values. This distribution indicated that a significant proportion of patients (31.0%) experienced ALT elevations 3–5 times higher than the reference values. Nearly a quarter of the patients maintained their ALT levels within the reference range, whereas another quarter experienced elevations 1–2 times above the reference values. Fewer patients had more frequent elevations, with 16.9% experiencing levels 6–10 times above the reference values and smaller percentages exceeding this range. Statistical analysis indicated that there was no significant association between the frequency of ALT levels being above the reference values and the clinical form (moderate or severe), based on the Pearson chi-square test (*p* = *0.227*) and the likelihood ratio test (*p* = *0.191*).

The mean disease day of hospitalization was higher for the severe clinical form of measles (5.13 days) than for the moderate clinical form (4.16 days). The 95% confidence intervals for the means did not overlap, suggesting a significant difference between them. The standard deviation was higher for the severe form (1.258 days) than for the moderate form (1.118 days), indicating a greater variability in the severe group. The skewness and kurtosis values indicated that the distribution of hospitalization days was slightly positively skewed for the moderate form and nearly symmetrical for the severe form. The moderate form had a more peaked distribution, whereas the severe form had a flatter one. Statistical analysis highlighted the presence of a significant linear association between the day of hospitalization and the clinical form (moderate or severe), based on the linear-by-linear association test (*p* = *0.005*). This was further supported by Pearson’s R and Spearman’s correlations, which both showed statistically significant moderate positive correlations (*p* = *0.004* and *p* = *0.006*, respectively). Although the Pearson chi-square (*p* = *0.094*) and likelihood ratio (*p* = *0.100*) tests did not show statistically significant associations at the 5% level, the close values suggested a potential trend.

In our study, we also assessed the prevalence of various symptoms, including diarrhea, nausea, vomiting, anorexia, pharyngitis, cephalalgia, asthenia, and dyspnea. The results revealed that the most frequently reported symptoms associated with measles were anorexia in 13 cases (18.3%), diarrhea in 8 cases (11.3%), and nausea also in 8 cases (11.3%). Less common symptoms included vomiting in four cases (5.6%), asthenia in six cases (8.5%), dyspnea in two cases (2.8%), pharyngitis and cephalalgia each in one case (1.4%). The majority of patients did not report these symptoms, ranging from 81.7% (58 cases) for anorexia to 98.6% (70 cases) for both pharyngitis and cephalalgia. These results indicated that while a subset of patients experienced significant symptoms, the overall prevalence of severe symptoms was relatively low.

Our cross-tabulations of different symptoms associated with the type of clinical form of the disease revealed no significant association and non-significant correlations between the clinical form and diarrhea, vomiting, anorexia, pharyngitis, cephalalgia, and asthenia in our sample of patients. Our results highlighted that there was a significant association between the occurrence of nausea and the clinical form (moderate or severe; 4 out of the 16 cases with a severe form of the disease reported nausea vs. 4 out of the 51 cases with a moderate form of disease), based on the Pearson chi-square test (*p* = *0.048*) and linear-by-linear association test (*p* = *0.050*). Pearson’s R and Spearman’s correlations were also statistically significant (*p* = *0.049*). Two patients reported dyspnea, and both presented with severe clinical forms of the disease. Our analysis suggested a significant association between the occurrence of dyspnea and the clinical form (moderate or severe), based on the Pearson chi-square test (*p* = *0.008*), likelihood ratio test (*p* = *0.013*), Fisher’s exact test (*p* = *0.048*), and linear-by-linear association test (*p* = *0.008*). Pearson’s R and Spearman’s correlations also indicated statistically significant moderate correlations (*p* = *0.007*). However, the continuity correction (*p* = *0.072*) did not show statistically significant results.

The most prevalent comorbidity condition among the patients in our study was high blood pressure, which affected four patients (5.6%), followed by sinus tachycardia, which affected five patients (7.0%), and chronic obstructive pulmonary disease (COPD), which also affected four patients (5.6%). Other conditions, such as pulmonary fibrosis, thrombophilia, diabetes mellitus, history of pulmonary tuberculosis, cholestasis, gastric ulcer, cachexia, primary immunodeficiency, asthma, and hypothyroidism, were present but less common, each affecting one patient (1.4%). Notably, pulmonary fibrosis, diabetes mellitus, and a history of pulmonary tuberculosis each affected two patients (2.8%). Obesity was the most common condition, affecting 12 (16.9%) enrolled patients.

Cross-tabulations between comorbidities and clinical form of the disease were also assessed. The only significant association was observed between the presence of COPD and its clinical form (moderate or severe). Pearson’s chi-square test (*p* = *0.010*), continuity correction (*p* = *0.049*), likelihood ratio test (*p* = *0.021*), and Fisher’s exact test (*p* = *0.034*) all indicated statistically significant associations. In addition, the linear-by-linear association test (*p* = *0.010*) supported these findings. Both Pearson’s R and Spearman’s correlations demonstrated statistically significant moderate positive correlations (*p* = *0.009*).

Regarding concomitant bacterial infections, seven patients presented with urinary tract infections (UTIs) caused by *Escherichia coli*, and one patient had a UTI caused by *Klebsiella pneumoniae*. Additionally, *Klebsiella oxytoca* and *Haemophilus influenzae* were isolated from the sputum of one patient. Methicillin-sensitive *Staphylococcus aureus* (MSSA) and methicillin-resistant *Staphylococcus aureus* (MRSA) were isolated from nasopharyngeal swabs in two cases. Furthermore, isolates of Group A and Group B *Streptococcus* were identified in one patient each from nasopharyngeal swabs. In addition, one patient presented with a concomitant upper respiratory tract infection with rhinovirus, and another presented with an infection caused by the herpes simplex virus.

## 4. Discussion

The decrease in measles vaccination rates in Romania was associated with measles epidemics, which primarily affected children until 2018. Post-COVID-19 pandemic, we are witnessing a new wave of measles epidemics that also affect adults, either young individuals vaccinated with only one dose, leading to a significant reduction in protection, or those born before 1979 (the year vaccination was introduced in Romania), who were not naturally immunized through the disease. Although the cold season is more frequently associated with the circulation of the measles virus, cases are present in this epidemic wave, even during the warm season.

The majority of hospitalized patients in our study were young, with an average age of 36.05 years for women and 32.21 years for men, and presented with multiple comorbidities, including cardiovascular and pulmonary conditions, diabetes mellitus, primary immunodeficiency, and obesity (16.9%). Fifteen patients presented with bacterial or viral co-infections (two cases), most frequently urinary and respiratory infections. From sputum cultures, *Klebsiella oxytoca* and *Haemophilus influenzae* were isolated, while *Staphylococcus aureus* was isolated in four cases from nasopharyngeal exudates; these etiologies were reported in literature to be possibly responsible for bacterial complications in measles [13].

The clinical picture also followed, in our study, the known stages, with onset marked by high fever, coryza, hyperlacrimation, and digestive manifestations, including anorexia, nausea, vomiting, and diarrhea. Nausea was significantly associated with severe forms of the disease. The exanthem appeared with generalization over three days, but erythematous-maculose elements were present on the limbs from the first day of evolution. Dyspnea was also significantly associated with severe forms of the disease. The evolution of the cases was favorable, with only one patient with morbid obesity, high blood pressure, COPD, and diabetes progressing to death. Additionally, COPD was significantly associated with severe forms of measles. During convalescence, a 23-year-old male presented with pneumomediastinum and subcutaneous emphysema, rarely described in literature [13].

In our cohort of patients, hepatic involvement in measles, manifested as hepatocellular injury, was present in 87.3% of cases, based on AST levels, and 76% had elevated ALT levels. The study revealed jaundice in 12.7% of patients with measles, three times more frequently than in the study by Dinh et al. [5], where hyperbilirubinemia was found in 4% of patients and elevated transaminases in two-thirds of cases. GGT changes were present in 35.2% of the cases, with 11.3% having values 3–5 times above the normal range. There was a statistically significant correlation between gamma-glutamyl transferase levels and the clinical form (moderate or severe). In addition, the results of our statistical analysis suggested a significant linear association between the neutrophil-to-lymphocyte (NL) ratio and clinical form (moderate or severe), based on the linear-by-linear association test. This was further supported by Pearson’s R and Spearman’s correlations, which both showed statistically significant correlations.

Our study has several limitations. As this was a retrospective study, it may not have provided a complete understanding or comprehensive overview of the situation. As this study was conducted at a single center, there may be some heterogeneity in the data, and the relatively small sample size limits its representation to our county and nearby areas, rather than a diverse range of hospitals nationwide. Additionally, the specific timeframe of the patient analysis introduces the possibility of residual confounding. Despite these limitations, the strengths of this study include the use of a comprehensive database that encompasses a broad range of clinical and analytical variables recorded at the time of hospital admission and throughout hospitalization, providing valuable insights into the clinical characteristics and outcomes of patients.

## 5. Conclusions

In summary, our study highlights the prevalence of cytolytic hepatic involvement in adult patients with measles, as evidenced by elevated levels of liver enzymes, such as AST and ALT. This type of hepatic involvement appears to be a common laboratory finding in most cases but does not correlate with the clinical severity of the disease. In contrast, cholestatic liver disease, characterized by elevated GGT levels, although less frequently observed, does show a significant association with more severe clinical forms of measles. These findings suggest that while cytolytic hepatic damage is a typical response to measles infection, cholestatic involvement may serve as an indicator of more severe disease progression. Further research is needed to explore these hepatic changes and to assess their potential impact on patient management and measles infection outcomes.

## Figures and Tables

**Table 1:** Characteristics of the study population.

Descriptive Statistics					Reference Levels
	Minimum	Maximum	Mean	Std. Deviation	
Aspartate aminotransferase (AST)	14	839	145.61	134.526	3–39 U/L
Alanine aminotransferase (ALT)	18	783	185.69	162.886	3–43 U/L
Gamma-glutamyl transferase (γGT)	12	1289	159.34	247.685	11–50 U/L
Total bilirubin	0.24	7.6	0.91	1.294	0.3–1.2 mg/dL
White Blood Cells (WBCs)	2350	15,900	5604.83	2719.784	4–10 × 10^3^/μL
Neutrophils	1100	10,040	4022.85	2109.219	2–7.5 × 10^3^/μL
Lymphocytes	260	2730	920.35	590.603	1.5–4 × 10^3^/μL
Neutrophil-to-Lymphocyte Ratio (NL Ratio)	0.71	14.93	5.7435	3.25734	
Disease day of hospitalization	2	7	4.38	1.211	
Demographic characteristics					
No. of enrolled patients	Study population		Male patients		Female patients
	71		34		37
Age (mean, std. dev)	34.21 years, 11.57 years		32.21 years 11.85 years		36.05 years, 11.15 years
Clinical manifestations at the time of admission					
	No. of patients (*n*=)				
Pyrexia	66				
Maculopapular exanthem	60				
Oculonasal catarrh	64				
Koplik’s spot	12				
Cough	64				
Pneumonia	61				
Clinical form	Moderate (55), Severe (16)				
Respiratory failure requiring different forms of oxygen therapy	18				
Diarrhea	8				
Nausea	8				
Vomiting	4				
Anorexia	13				
Asthenia	6				
Dyspnea	2				
Pharyngitis, Cephalalgia	1				

## Data Availability

The data presented in this study are available upon reasonable request from the corresponding author.

## References

[1] Berry T.J. (1960). Hepatic Damage Associated with Measles. Pa. Med. J..

[2] Gavish D. (1983). Hepatitis and Jaundice Associated with Measles in Young Adults. Arch. Intern. Med..

[3] Giladi M., Schulman A., Kedem R., Danon Y.L. (1987). Measles in Adults: A Prospective Study of 291 Consecutive Cases. BMJ.

[4] Leibovici L., Sharir T., Kalter-Leibovici O., Alpert G., Epstein L.M. (1988). An Outbreak of Measles among Young Adults. Clinical and Laboratory Features in 461 Patients. J. Adolesc. Health Care.

[5] Dinh A., Fleuret V., Hanslik T. (2013). Liver Involvement in Adults with Measles. Int. J. Infect. Dis..

[6] Biron C., Beaudoux O., Ponge A., Briend-Godet V., Corne F., Tripodi D., Hazart I., Esbelin J., Biron A., Boutoille D. (2011). Rougeole Au CHU de Nantes Au Cours de l’épidémie 2008–2009. Med. Mal. Infect..

[7] Sternfeld T., Spöri-Byrtus V., Riediger C., Langer R., Friess H., Schmid R.M., Schulte-Frohlinde E. (2010). Acute Measles Infection Triggering an Episode of Liver Transplant Rejection. Int. J. Infect. Dis..

[8] Satoh A., Kobayashi H., Yoshida T., Tanaka A., Kawajiri T., Oki Y., Kasugai K., Tonai M., Satoh K., Nitta M. (1999). Clinicopathological Study on Liver Dysfunction in Measles. Intern. Med..

[9] Nobili V., Pietro S., Stefania P. (2007). Fulminant Hepatic Failure Following Measles. Pediatr. Infect. Dis. J..

[10] Sati S.K., Banga S., Bhadouria S.S. (2018). Fulminant Hepatic Failure in Measles in a 6-Month-Old Child. Int. J. Clin. Pediatr..

[11] Eto Y., Terao H., Shigeno H., Tashiro T., Fujioka T., Nasu M. (1991). A Clinical Study on Liver Dysfunction in Patients with Acute Measles Infection. J. Jpn. Assoc. Infect. Dis..

[12] Institutul National de Sanatate Publica Situatia Rujeolei in Romania. https://insp.gov.ro/downloads/rujeola/.

[13] Loukides S., Panagou P., Kolokouris D., Kalogeropoulos N. (1999). Bacterial Pneumonia as a Suprainfection in Young Adults with Measles. Eur. Respir. J..

